# Axonal injuries and consequences to neuronal computation

**DOI:** 10.1186/1471-2202-14-S1-P231

**Published:** 2013-07-08

**Authors:** Pedro D Maia, J Nathan Kutz

**Affiliations:** 1 Department of Applied Mathematics, University of Washington, Seattle, WA, 98105, USA

## 

Axonal injury and swelling is a common outcome of brain trauma and/or concussion. Recent experimentally observed axonal deformations induce irregularities in the axonal morphology and geometry [1,2]. Such irregularities, combined with nonlinear gating properties of ion channels, can significantly alter firing rate patterns between the initiation and arrival of spikes at pre-synaptic sites. As a consequence, information encoded in spike trains propagating in the axon can be jeopardized due to injury.

**Figure 1:**
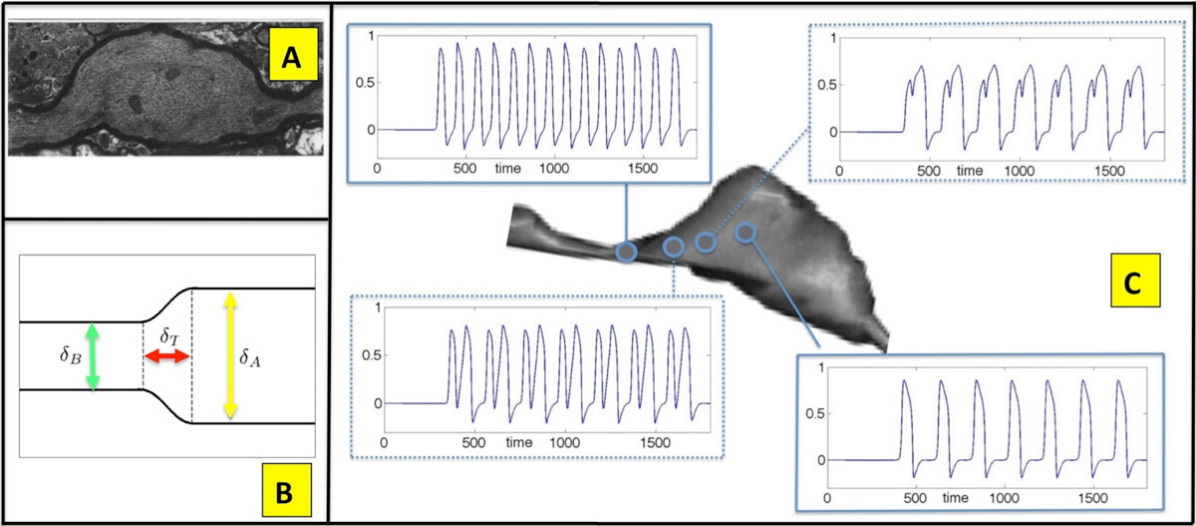
(A) Axonal swelling due to injury. (B) Model of enlargement. (C) Axonal geometry reshaping spike trains.

We develop a computational framework to evaluate and distinguish between axonal injuries that lead to geometrical enlargements responsible for producing minor changes in propagation from those that result in critical phenomenon such as reflection or blockage of the original traveling spike train. We use a few geometrical parameters to model a prototypical axon enlargement and explore numerically the parameter space characterizing all possible propagation regimes and dynamics in an unmylienated action potential model. Figure 1 demonstrates an observed axon swelling [Figure 1A] a prototypical model [Figure 1B] along with its impact on a typical spike train [Figure 1C]. The geometry changes induced by the swelling can lead to significant change in the spike train dynamics and timing, thus jeopardizing faithful conductance of information.

Contrary to earlier notions that large diameter increases mostly lead to blocking, we demonstrate transmission is stable provided the geometrical changes occur in a slow (adiabatic) manner. As a consequence, swellings of lesser volumes but with abrupt increase in diameter can be more dangerous than those of larger volumes with adiabatic changes in geometry. Our method also identifies a narrow range of parameters leading to a reflection regime. The distinction between these three regimes can be evaluated by a simple analytical function of the geometrical parameters inferred through numerical simulations. Additionally, the effect on information encoding can be evaluated in such injured axons by performing a spike metric analysis [3]. Given that the statistics of both swelling size and frequency are known from recent experiments [1, 2, and references therein], our method allows or a prediction of the loss of neural computation due to traumatic brain injury and/or concussion.
